# On the Treatment of the Laceration of the Perineum in Parturition

**Published:** 1820-09

**Authors:** Thomas Alcock

**Affiliations:** Member of the Royal College of Surgeons; Accoucheur to the St. James's Infirmary, &c.


FOR THE LONDON MEDICAL AND PHYSICAL JOURNAL.

### *On the Treatment of the Laceration of the Perineum in Parturition.*

By Thomas Alcock, Member of the Royal College of Surgeons; Accoucheur to the St. James's Infirmary, &c.

I VENTURE to submit the following remarks through the medium of your Journal, in the hope that the principle of treatment which was successfully employed in the case alluded to below, may be useful to others, as I am not aware that the plan proposed has been hitherto adopted.

From a series of observations during several years, and from my official duties, which render the field of observation more extensive than falls to the lot of many practitioners, I am persuaded that laceration of the perineum, although it very rarely happens under skilful management, is a much more frequent accident than it is generally supposed to be; particularly amongst women attended by midwives, who often endeavour to expedite the delivery with more zeal than sound judgment.

The consequences of laceration of the perineum to any considerable extent are often very grievous, both as affecting the personal comfort of the individual, and not unfrequently as laying the foundation of family dissensions, which cannot be too deeply regretted.

Several years ago I witnessed a most distressing case of laceration of the perineum, produced (according to the patient's statement) by instrumental delivery in a public Lying-in Hospital. For the honour of the profession, I must believe that this patient's statement was incorrect; but that she was an extreme sufferer, was beyond all doubt. The vulva, vagina, perineum, and lower part of the rectum, were torn into one hideous gap, into which the urine constantly dribbled and mixed with the feces. Sloughing took place to such an extent, that for several weeks her chance of being able to drag on a miserable existence was extremely doubtful; whilst her sufferings were so great, that her mother and others were used to ejaculate, "What a mercy it would be if it would please God to take her!"

Procidentia uteri, and, in its worst form, prolapsus, is frequently a consequence of laceration of the perineum. It is a well-known fact to those conversant in midwifery, that the ligaments of the uterus, elongated as they are by the increase of size of the uterus during gestation, are not sufficient to prevent the falling-down of that organ after delivery, if the erect position, or strong bodily exertion, be permitted before the uterus has regained its natural dimensions, and before the vagina has recovered from the extreme dilatation it has suffered during labour. Hence the falling-down of the uterus below its natural situation, is a common occurrence, and this tendency is increased by every subsequent labour; so much so, that it may be assumed, that, among females who obtain their living by laborious exertion, there is scarcely a mother of a family who is wholly free from this malady. It is frequently brought on by the want of proper knowledge, or by impatience in early rising after delivery: more generally, however, amongst the industrious classes, these circumstances take place from necessity. I have known the mother of a young family in humble life, unable to defray the expense of a nurse, rise from child-bed on the third day, and resume her domestic employments; a procedure which, if it be not productive of immediate illness, seldom fails to produce injury at a more distant period.

Under the circumstances above mentioned, and in the first stage of the complaint, the uterus falls down into the vagina, resting upon the posterior part of the latter, and supported by the perineum; a state which, although attended by leucorrhœa and pain and weakness in the loins, is often submitted to without complaint. But if, in addition to the causes above enumerated, the perineum be lacerated, so as to be incapable of affording its usual support, the uterus does not remain within the vagina, but gradually descends beyond the external parts, and in extreme cases inverts the vagina, which thus serves as an external covering. The want of support from the perineum also prevents the use of pessaries, except those of sponge, which will not in all cases afford the palliation required.

A case of this nature came under my observation in the year 1811, in which the uterus was pendulous, and enlarged to the size of a child's head, hanging entirely beyond the external parts. The os uteri was the most depending point, and the inverted vagina formed the external covering of the tumor. The greater part of the inverted vagina had acquired much of the appearance of the common integument; but the lower part, or that attached to the os uteri, was ulcerated to the extent of several fingers' breadth, taking a lateral direction. The ulcerated surface was red and glassy, and had not for years shown any disposition to heal, probably owing to the constant friction and irritation occasioned by the patient's clothes. She had laboured under the disease during the last fourteen years; in the early part of which she had first noticed the obstruction in the vagina, which increased after delivery, particularly when she was employed in carrying heavy weights; and the external tumor gradually succeeded and increased in bulk, till it had attained the size before stated. She had been a patient of various hospitals and dispensaries, but without relief. She could not, from the friction on the ulcerated surface, wear a bandage; neither had the use of pessaries, lotions, &c. improved her loathsome condition. There was a copious and offensive discharge, but whether from the ulcerated surface or from the mouth of the womb, she could not determine, probably from both; and the frequent and urgent call to pass urine, with the almost constant presence of diarrhœa, rendered her miserable. She could return the tumor within the pelvis without difficulty when in the recumbent posture, (for the opening was sufficiently large,) or, by considerable effort, by bending the body forwards, and pressing the tumor upwards, but it descended again as soon as she resumed the erect position.

She stated that, five years before this period, the tumor had swelled so much, that for many days it could not be returned; but that it had remained reducible ever since.

Distressing as this case certainly was, and although, from its duration, the idea of cure could not be entertained, yet I am inclined to believe that, had the re-union of the perineum been effected, she might have been materially relieved, so as to render her forlorn condition at least tolerable.

The following case was widely different in result from the former:

Mrs. T. aged twenty-three years, at the full period of her first pregnancy, was delivered of a large still-born infant; but, owing to a mistake in the address given, she was without professional assistance at the moment the child was born. A laceration of the perineum, through its whole extent to within half an inch of the anus, was the consequence. She complained greatly of the pain of the external parts, which were much swollen, and of a deep-red colour. Two days after delivery it was evident that the lacerated edges would slough; and a thin surface was thrown off at the end of a week, leaving the ulcerated parts clean and florid.

Aware that the general usage is to leave such cases to nature, and that the attempt to unite the lacerated perineum surgically is not sanctioned by public teachers, I could not however see any solid reason why this patient should be left without an attempt to remedy the injury she had sustained; as, should it fail, she would not be in a worse situation than before. The nature of the case was candidly stated to the patient, and the means to be used explained. She was willing to undergo any operation I might deem necessary.

The ulcerated edges were accurately placed in apposition, and were retained by a suture passed midway between the extremities of the lacerated portions: the knees were kept together by a slight bandage, and strict attention to cleanliness was enjoined; the discharges being prevented from lodging in the vagina by the frequent use of tepid milk and water as an injection.

In three days the ligature was removed, and union from the point where the suture was inserted was perfect posteriorly; but it had not, as was anticipated, extended anteriorly to the suture. Another ligature was therefore passed within a few lines of the most anterior point of ulceration, and in three days more the union was complete, and the perineum was restored to the same state as before the accident.

The progress of this case was witnessed by the respectable Editor of the Medical Intelligencer, and other of my professional friends, as well as by my pupils.

I saw this patient several months afterwards: she was in good health; and she informed me that she had remained as well, and as perfectly free from inconvenience, as at any period previous to her confinement.

The result of this case proved highly satisfactory to the patient, and to her immediate relations. The treatment was unattended with danger, and the inconvenience was much less than the usual cicatrization of such an extent of lacerated surface; so that the advantage gained will scarcely admit of doubt.

Should I again have occasion to perform this operation, I would however place the ligatures nearer to each other than is usual on common occasions, taking care that the anterior point should be well supported; as the natural tendency of a sphincter muscle would cause retraction and prevent union. I am also persuaded that the mode of operating for the hare-lip might be applicable to old and neglected cases of lacerated perineum; whilst the means used for hare-lip, with such adaptations and precautions as would suggest themselves to the intelligent practitioner, would possess some advantages beyond those which were employed in the case which led to these remarks.

3, *Piccadilly; August* 1820.

